# Crystal Structure of USP7 Ubiquitin-like Domains with an ICP0 Peptide Reveals a Novel Mechanism Used by Viral and Cellular Proteins to Target USP7

**DOI:** 10.1371/journal.ppat.1004950

**Published:** 2015-06-05

**Authors:** Roland Pfoh, Ira Kay Lacdao, Anna A. Georges, Adam Capar, Hong Zheng, Lori Frappier, Vivian Saridakis

**Affiliations:** 1 Department of Biology, York University, Toronto, Ontario, Canada; 2 Department of Molecular Genetics, University of Toronto, Toronto, Ontario, Canada; Washington University, UNITED STATES

## Abstract

Herpes simplex virus-1 immediate-early protein ICP0 activates viral genes during early stages of infection, affects cellular levels of multiple host proteins and is crucial for effective lytic infection. Being a RING-type E3 ligase prone to auto-ubiquitination, ICP0 relies on human deubiquitinating enzyme USP7 for protection against 26S proteasomal mediated degradation. USP7 is involved in apoptosis, epigenetics, cell proliferation and is targeted by several herpesviruses. Several USP7 partners, including ICP0, GMPS, and UHRF1, interact through its C-terminal domain (CTD), which contains five ubiquitin-like (Ubl) structures. Despite the fact that USP7 has emerged as a drug target for cancer therapy, structural details of USP7 regulation and the molecular mechanism of interaction at its CTD have remained elusive. Here, we mapped the binding site between an ICP0 peptide and USP7 and determined the crystal structure of the first three Ubl domains bound to the ICP0 peptide, which showed that ICP0 binds to a loop on Ubl2. Sequences similar to the USP7-binding site in ICP0 were identified in GMPS and UHRF1 and shown to bind USP7-CTD through Ubl2. In addition, co-immunoprecipitation assays in human cells comparing binding to USP7 with and without a Ubl2 mutation, confirmed the importance of the Ubl2 binding pocket for binding ICP0, GMPS and UHRF1. Therefore we have identified a novel mechanism of USP7 recognition that is used by both viral and cellular proteins. Our structural information was used to generate a model of near full-length USP7, showing the relative position of the ICP0/GMPS/UHRF1 binding pocket and the structural basis by which it could regulate enzymatic activity.

## Author Summary

USP7 is a cellular protein that binds and stabilizes many proteins involved in multiple pathways that regulate oncogenesis and as such is recognized as a potential target for cancer therapy. In addition, USP7 is targeted by several viral proteins in order to promote cell survival and viral infection. One such protein is the ICP0 protein of herpes simplex virus 1, which must bind USP7 in order to manipulate the cell in ways that enable efficient viral infection. Here we use a structural approach to define the mechanism of the USP7-ICP0 peptide interaction, revealing a novel binding site on USP7. We then used this information to identify two cellular proteins, GMPS and UHRF1, that also bind USP7 through this binding site. Therefore we have identified a new mechanism by which both viral and cellular proteins can target USP7. This information will be useful for the development of strategies to block specific protein interactions with USP7.

## Introduction

Ubiquitin specific protease 7 (USP7) catalyzes the deubiquitination of many cellular proteins involved in tumor suppression, neural stem cell maintenance, DNA damage and immune responses [1–8]. USP7 consists of an N-terminal TRAF-like domain (NTD), a catalytic domain (CAT) and five C-terminal ubiquitin-like domains (CTD). Many USP7 interaction partners bind to a shallow groove on the surface of USP7-NTD using a P/A/ExxS motif including p53, Hdm2, HdmX, UbE2E1, MCM-BP, Epstein-Barr virus (EBV) protein EBNA1 and Kaposi’s sarcoma associated herpesvirus (KSHV) protein vIRF4 [9–13]. Some USP7 interacting proteins bind to USP7-CTD including the ICP0 protein of herpes simplex virus 1 (HSV-1), GMP synthase (GMPS), and UHRF1, however their molecular mechanisms of interaction have not been extensively characterized [14–16]. The crystal structure of the USP7-CTD revealed a 12-3-45 Ubl domain architecture with di-Ubls formed between the first (Ubl12) and the last two (Ubl45) domains [15]. In contrast, Ubl3 displays limited contacts [15]. Affinity chromatography coupled with *in vitro* proteolysis revealed that a region within residues 560–870, which corresponds to Ubl123, interacts with ICP0 [14]. Similarly, Ubl123 has been reported to interact with GMPS [15]; a metabolic enzyme involved in nucleotide biosynthesis with a second independent function as a USP7 modulator [17–19]. GMPS enables USP7-dependent deubiquitination of histone H2B resulting in epigenetic silencing [17–19]. GMPS also enhances the USP7 catalyzed deubiquitination of p53 [19, 20]. The ability of GMPS to activate ubiquitin cleavage by USP7 involves its interaction with USP7-CTD, which is thought to stabilize a compact USP7 conformation leading to ordering of active site residues and stimulation of catalytic activity [15].

Another important USP7-CTD interacting protein is the epigenetic regulator UHRF1 (also known as NP95), an E3 ligase which recognizes hemi-methylated DNA on newly replicated strands and recruits DNMT1, a DNA methyltransferase, to methylate these CpG sites [1, 21]. Both UHRF1 and DNMT1 are deubiquitinated and stabilized by USP7 [22, 23], while UHRF1 is the negative regulator of DNMT1 [1]. Interestingly, UHRF1 interacts with both the N and C terminal domains of USP7 [16, 22], similar to other substrates including p53 and Hdm2 [24].

Several proteins from herpesviruses target USP7 predominantly to undermine cellular defense mechanisms [9, 25–27]. In fact USP7 was first discovered through its strong and specific interaction with the ICP0 protein of HSV-1, leading to its original name of Herpesvirus Associated USP (HAUSP) [27]. Studies performed with ICP0-null mutants demonstrated that ICP0 is important for viral replication, as these viruses had impaired growth [28]. ICP0 is an E3 ubiquitin ligase with important roles in HSV-1 lytic infection and in reactivation of the virus from the latent state [29, 30]. Via its E3 ligase function, ICP0 promotes the ubiquitination and degradation of multiple cellular proteins, including promyelocytic leukemia proteins (PML), Sp100, DNA-PKcs, RNF8 and RNF168, thereby overcoming the intrinsic antiviral response [31–34]. Like most other RING E3 ligases, ICP0 auto-ubiquitinates and targets itself for proteasome mediated degradation. USP7 stabilizes ICP0 by interfering with its self-ubiquitination activity [35]. Conversely ICP0 induces the degradation of USP7 [36]. Studies on the mechanism of the USP7-ICP0 interaction showed that ICP0 residues 594–633, and K620, in particular, are important for USP7 binding [37, 38]. ICP0 mutant viruses lacking the USP7 binding site grow poorly, indicating that the ICP0-USP7 interaction is crucial for an effective HSV-1 infection [38, 39]. It has also been reported that ICP0 can translocate USP7 from the nucleus to the cytosol, where USP7 in complex with ICP0 attenuates the toll-like receptor (TLR) regulated immune response by deubiquitinating TRAF6 and IKKγ (also known as NEMO) [40].

Despite the importance of USP7 and its growing substrate list, molecular details of regulation of its catalytic activity are not yet well understood. Different ternary USP7 complexes involving USP7-GMPS-H2B, USP7-GMPS-p53 and USP7-UHRF1-DNMT1, have been reported, but so far only a single N-terminal binding site has been described for USP7 [17, 19, 20, 22]. The fact that several proteins bind to the C-terminal ubiquitin-like domains of USP7 prompted us to identify and characterize the interaction mechanism. This study shows the structural basis of interaction between the first three C-terminal ubiquitin-like domains complexed with an ICP0 peptide, revealing a protein binding pocket at Ubl2 of USP7-CTD. The interacting site in ICP0 was used to identify a similar motif in other USP7-binding proteins, leading to the identification of GMPS and UHRF1 as proteins that also bind the Ubl2 binding pocket. Therefore, we have identified a novel C-terminal domain mechanism of USP7 binding that is utilized by both viral and cellular proteins.

## Results

### Mapping the USP7 binding region on ICP0

**Fig 1:**
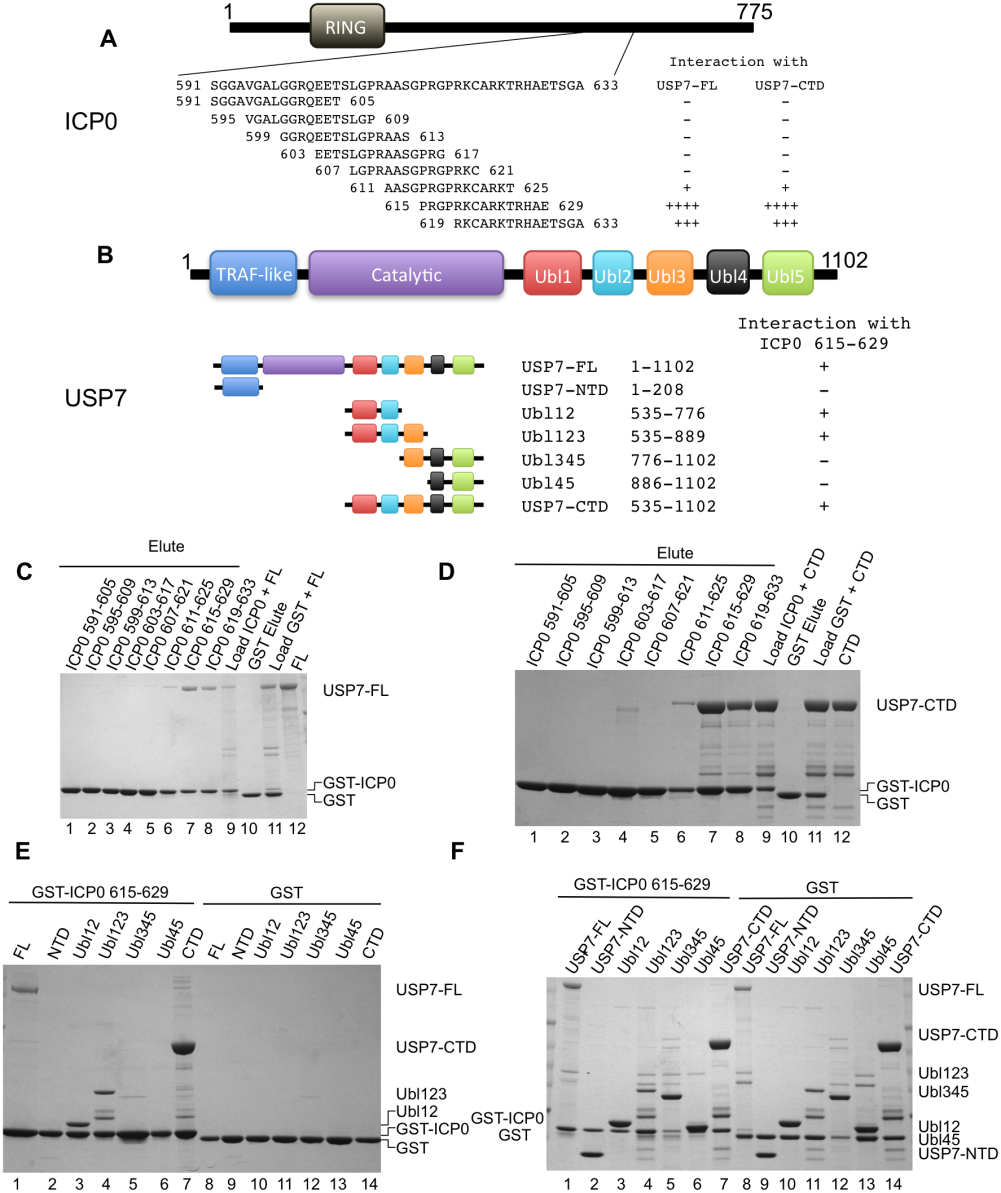
Ubl123 interacts with ICP0 residues 615–629. (A) ICP0 domain structure and overlapping peptide sequences. (B) USP7 domain structure and residues of the fragments. (C) Coomassie stained gel of GST pull-down assays between ICP0 peptides and USP7-FL. Lanes 1–8 are the eluted fractions (2.5% on gel). Lane 9 contains a sample of the load (USP7-FL and GST-ICP0 peptide, 1% on gel). Lanes 10 (elute) and 11 (load) are the GST only controls. Lane 12 is USP7-FL alone. (D) Coomassie stained gel of GST pull-down assays between ICP0 peptides and USP7-CTD. Lanes 1–8 are the eluted fractions (2.5% on gel). Lane 9 contains a sample of the load (USP7-CTD and GST-ICP0 peptide, 1% on gel). Lanes 10 (elute) and 11 (load) are the GST only controls. Lane 12 is USP7-CTD alone. (E) Coomassie stained gel of GST pull-down assays between ICP0 residues 615–629 and various USP7 domains. Lanes 1–7 are the eluted fractions with USP7-FL (15%), USP7-NTD (2.5%), Ubl12 (2.5%), Ubl123 (2.5%), Ubl345 (10%), Ubl45 (2.5%) and USP7-CTD (2.5%). Lanes 8–14 are the GST only controls. (F) Coomassie stained gel of loads used in (E). Approximately 1% of the input is loaded on the gel.

The USP7 binding region is found within ICP0 residues 594–633 and mutagenesis studies have shown that K620 is essential for the interaction [38]. In order to facilitate crystallization and structure determination, we set out to identify a shorter peptide that is capable of binding to USP7. To this end, a series of overlapping GST-ICP0 fusions (591–605, 595–609, 599–613, 603–617, 607–621, 611–625, 615–629, 619–633) were prepared (see Fig 1A for sequence details) and used in GST pull-down assays. GST pull-down assays with FL-USP7 and USP7-CTD (see Fig 1B for sequence details) identified that ICP0 peptides 615–629 and 619–633 show the strongest interaction with USP7 (Fig 1C and 1D). ICP0 region 611–625 also interacted with FL-USP7 and USP7-CTD but not to the same extent as residues 615–629 or 619–633 (Fig 1C and 1D). The remaining GST-ICP0 fusions did not show detectable interactions with FL-USP7 and USP7-CTD. GST alone was used as the negative control and did not show detectable binding to either FL-USP7 or USP7-CTD. Overall, our GST pull-down assays identified ICP0 residues between 619 and 629 as important mediators of USP7 binding in agreement with a previous study [38].

Next we determined which region of USP7-CTD interacted with the ICP0 615–629 peptide. FL-USP7, USP7-CTD as well as Ubl12, Ubl123, Ubl345 and Ubl45 (Fig 1B) were used as prey in GST pull-down assays using GST-ICP0 615–629. FL-USP7 and USP7-CTD were used as positive controls while GST alone and USP7-NTD were used as negative controls. The GST pull-down assays showed that FL-USP7 as well as USP7-CTD but not USP7-NTD or GST alone interacted with GST-ICP0 615–629, confirming that USP7-CTD interacts with this region of ICP0 (Fig 1E). Of the various CTD truncations at the ubiquitin-like domain boundaries, those containing Ubl12 (including Ubl12 and Ubl123) interacted with ICP0 peptide 615–629 (Fig 1E), suggesting that interaction with this ICP0 peptide is contained within Ubl12. There was no interaction seen with Ubl345 and Ubl45, again suggesting that the interaction occurring with Ubl12 and Ubl123 is specific (Fig 1E). Examination of the loads used in these assays (Fig 1F) show that the preference for binding Ubl12 and Ubl123 was not due to higher protein levels.

### Molecular recognition of ICP0 by USP7

**Fig 2:**
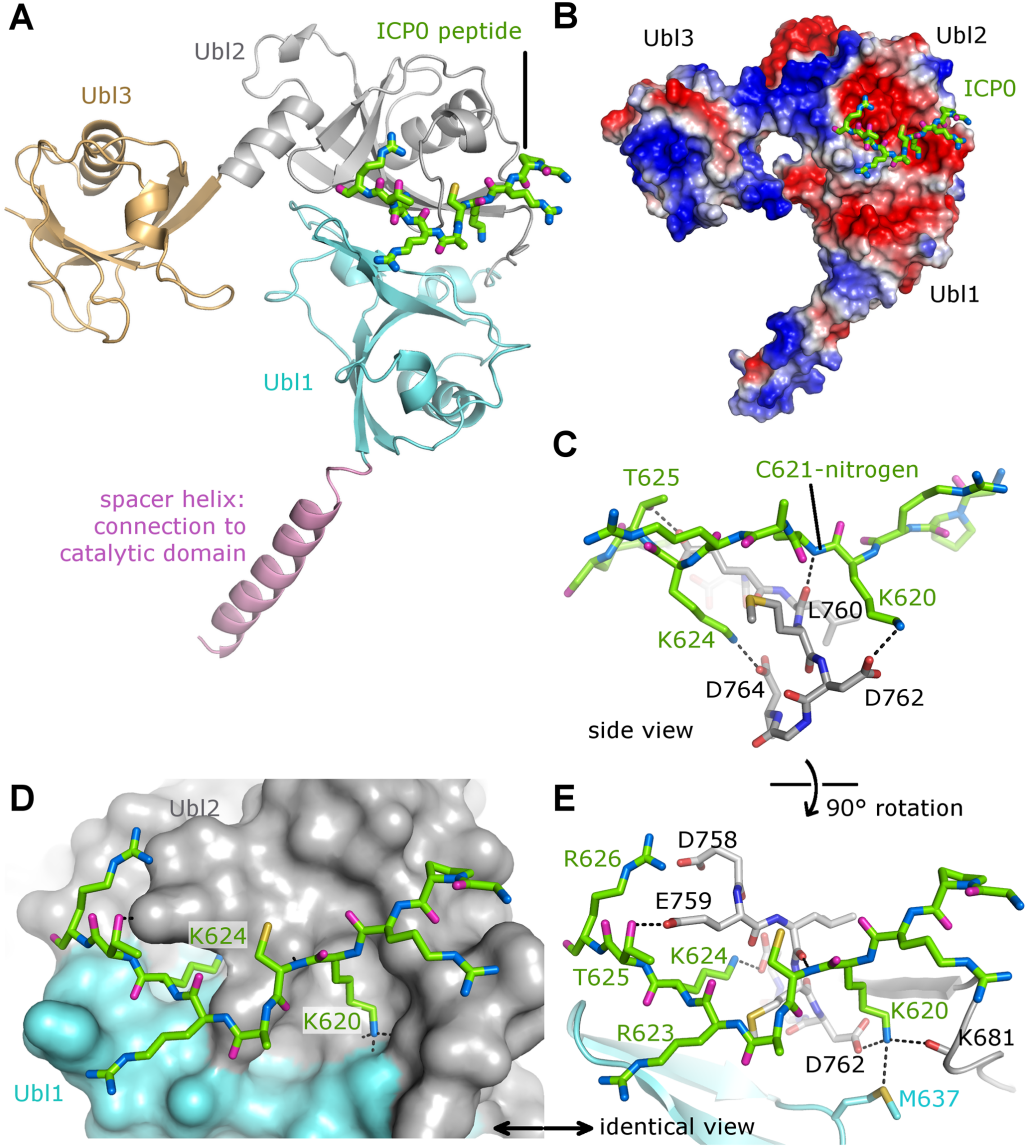
The crystal structure of the Ubl123-ICP0 peptide complex. (A) Interaction between Ubl123 and ICP0 peptide. Ubl123 is shown in cartoon representation and the ICP0 peptide in stick representation. (B) Electrostatic surface representation with acidic regions shown in red and basic regions in blue. (C) Front view of the ICP0 binding site. Interactions between Ub123 and ICP0 are highlighted by dashed lines. (D) Surface representation of the binding pocket with stick model of ICP0. (E) Side view of the ICP0 binding site. Interactions between Ub123 and ICP0 are highlighted by dashed lines.

To reveal the molecular basis of the interaction between USP7 and ICP0, ICP0 peptides ^617^GPRKCARKTRH^627^ or ^615^PRGPRKCARKTRHAE^629^ were used in co-crystallization trials with Ubl12 and Ubl123. Crystals were only obtained with Ubl123 and ICP0 peptide ^617^GPRKCARKTRH^627^. The crystal structure of Ubl123-ICP0 peptide complex was determined to 2.9 Å resolution (Table 1). The ICP0 peptide binds to a predominantly negatively charged area between ^758^DELMDGD^764^ on Ubl2 (Fig 2A and 2B). The two ICP0 lysine residues (K620 and K624) function like a pair of tongs by forming salt-bridges with two buried aspartate residues of USP7 (D762 and D764) (Fig 2C and 2D). These two lysine residues belong to the central part of the peptide (KCARKT), which is in close contact with USP7 due to a backbone-to-backbone hydrogen bond (ICP0 nitrogen-C621 with USP7 oxygen-L760, Fig 2D). In addition, threonine residue T625 of the peptide is forming a hydrogen-bond with USP7-D764 (Fig 2E). Only the ICP0 R623 side chain is pointing away from USP7 and is not involved in any binding interaction (Fig 2E). The central part of the peptide shows identical binding to both USP7 chains in the asymmetric unit and has a strong and clearly interpretable electron density (S1 Fig). Two peripheral arginine residues (R619 and R626, Fig 2E) positioned at both ends of the peptide are in proximity to aspartate residues positioned on the surface of USP7 (D682 and D758), but the distances indicate that these interactions are weaker than the ones involving K620 and K624. These arginine residues may interact with D682 and D758 in the context of full-length ICP0 protein interaction with USP7. In our crystal structure, the ^617^GPRKCARKTRH^627^ peptide binds most specifically to Ubl2 via its core KCARKT motif. The highly positive charge of the interacting ICP0 stretch might be important for the initial and more distant electrostatic interaction with the highly electronegative USP7 binding pocket (Fig 2B). ICP0 residue K620 interacts with M637 (Ubl1), D762 (Ubl2), and K681 (connecting loop between Ubl1 and Ubl2). The apo-structure of Ubl12 [15] is very similar to the peptide bound form (S2A and S2B Fig), with only a handful of side-chains in different rotamer conformations. Residues E759 and M637 change their side-chain conformations to form hydrogen-bonds with the peptide, whereas residues R628 and D754 move out to avoid clashes (S2C and S2D Fig). The similarity between apo- and peptide bound forms suggests that the ICP0 peptide binds to USP7 via a structurally stable pocket on Ubl2.

**Table 1:** Crystallographic statistics.

	native Ubl123	SeMet-Ubl123 (high-resolution)	SeMet-Ubl123 (peak)	SeMet-Ubl123 (inflection)
Protein conformation	compact	extended	extended	extended
PDB ID	4WPH	4WPI	-	-
Bound ligand	ICP0-peptide	ICP0-peptide	ICP0-peptide	ICP0-peptide
**Crystal data**				
Spacegroup	P3_2_21	P4_2_2_1_2	P4_2_2_1_2	P4_2_2_1_2
Unit cell (a = b, c) (Å)	92.33, 190.28	164.99, 110.12	165.03, 110.14	165.03, 110.14
**Diffraction data**				
Wavelength (Å)	1.5418	0.97849	0.97849	0.97871
Resolution limit (Å)	2.9	3.4	3.6	3.6
Data redundancy	1.9 (1.9)	9.5 (10.0)	9.4 (10.0)	9.6 (10.2)
R-merge (%)	3.4 (21.3)	6.88 (48.9)	6.6 (44.6)	6.0 (43.8)
I / (I)	11.5 (2.5)	24.3 (4.5)	27.0 (4.1)	19.7 (3.5)
Completeness (%)	99.5 (100.0)	99.7 (99.9)	99.7 (99.9)	99.8 (99.9)
**Model refinement**				
Unique reflections used	19 939	20 318		
Resolution range (Å)	30.0 - 2.92	30.0 - 3.40		
R*-*work (%)	23.3	24.1		
R*-*free (%)	26.2	28.2		
Ramachandran (Outlier/Allowed/Preferred residues)	0/15/651	2/60/629		
Water molecules	38	0		
Rmsd bond lengths (Å)	0.009	0.010		
Rmsd bond angles (°)	1.29	1.39		

Values in parentheses correspond to the highest resolution shell.

**Fig 3:**
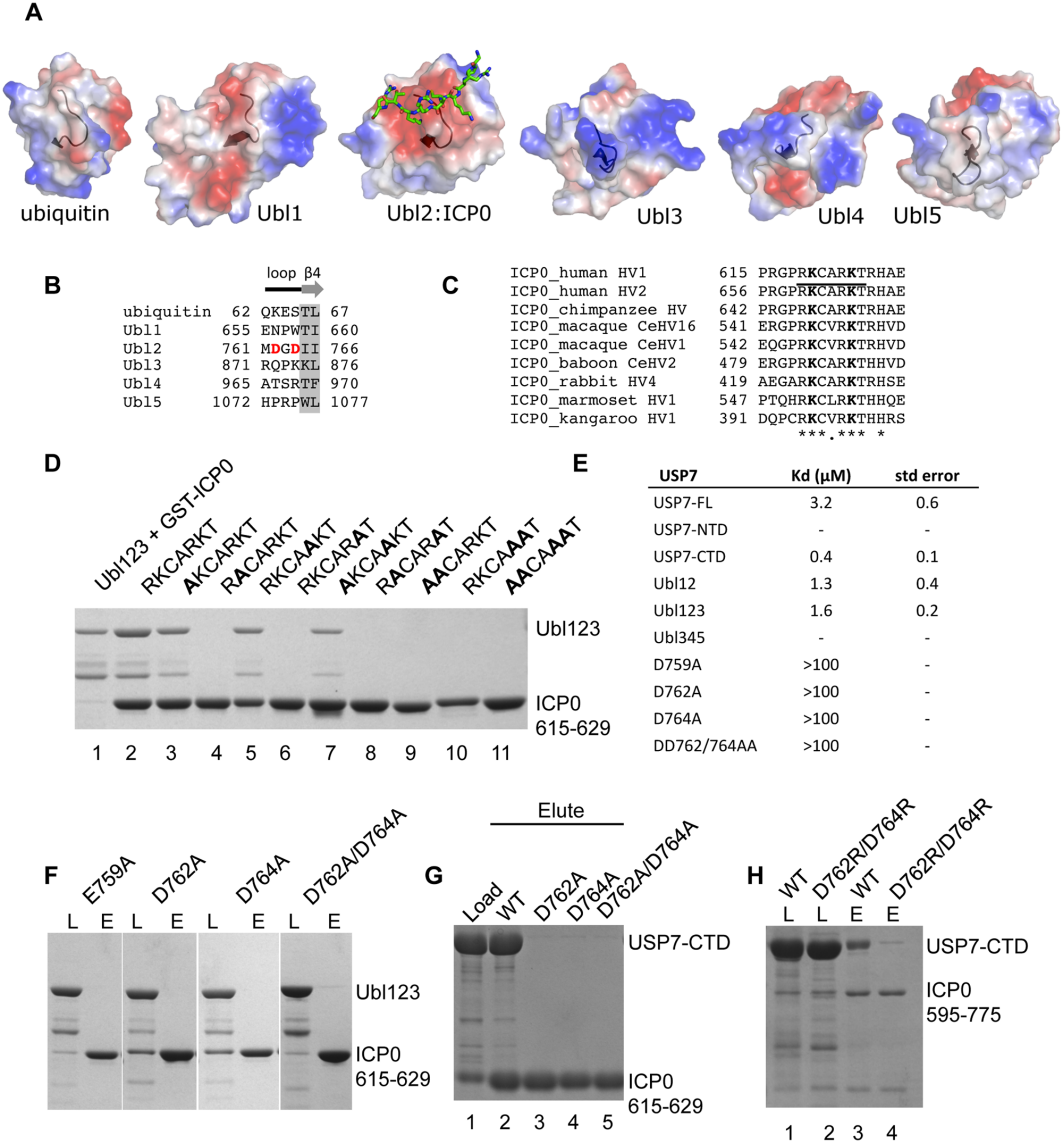
Ubl123 E759, D762 and D764 are essential for interaction with ICP0. (A) Electrostatic surface comparison of the Ubl domains. Ubiquitin and the five Ubls are shown in the same orientation, a black ribbon indicates the loop region ahead of the β4-strand. (B) Structure based sequence alignment of the ICP0 binding region involving key residues D762 and D764 in Ubl2. Since the loop structure and length varies in the five Ubls, only the residues directly neighboring the structurally conserved β4-strand were included. (C) Sequence alignment of ICP0 proteins from human HSV-1 (P08393), human HSV-2 (P28284), chimpanzee HV (K9MG59), macaque HV-16 (X2FDL5), macaque HV-1 (Q7T400), baboon HV-2 (Q5Y0P4), rabbit HV-4 (J9QWJ6), marmoset HV-1 (E2IUH0), and kangaroo HV-1 (Q91CH9). (D) Coomassie stained gel of GST pull-down assays with mutant GST-ICP0 peptides. Lanes 1 (load) and 2 (elute) Ubl123 and GST-ICP0, lanes 3–11 are the eluted fractions with GST-ICP0 mutants. (E) The dissociation constants for the USP7 interaction with FITC-labeled ICP0 peptide. Average values with standard deviation for three or more experiments are shown. (F) GST pull-down assays with mutant Ubl123 (E759A, D762A, D764A and D762A/D764A) and GST-ICP0 peptide. Load (L) and eluate (E) represent the loaded and eluted fractions of each Ubl123 mutant. (G) GST pull-down assays with WT or mutant USP7-CTD (D762A, D764A and D762A/D764A) and GST-ICP0 peptide. Lane 1 USP7-CTD and GST-ICP0 load, lanes 2–5 are the eluted fractions with WT and mutant USP7-CTD. (H) GST pull-down assays with WT or D762R/D764R USP7-CTD and GST-ICP0 594–775. Lanes 1 and 2 are loaded (L) samples. Lanes 3 and 4 are the eluted (E) fractions with WT and D762R/D764R USP7-CTD. In all instances, approximately 1% of the input and 2.5% of the eluate is loaded on the gels.

Each Ubl domain of USP7 adopts the characteristic ubiquitin-like ββαββ-fold [15]. The binding site for ICP0 (^758^DELMDGD^764^) is located on Ubl2 in a loop right ahead of the last β-strand (β4) of this ubiquitin fold. The sequence identities between the five ubiquitin-like domains are less than 15% and the length of the loop between α2 and β4 varies in the five Ubls [15]. The acidic patch surrounding the loop is only fully present in Ubl2 (Fig 3A) and a structure based alignment of the loop shows that only Ubl2 contains the key residues for ICP0 binding (Fig 3B).

### Identifying residues critical for the USP7-ICP0 interaction

Our structural analysis is consistent with a mutational study on ICP0 [38] reporting abolished binding to USP7 of ICP0 mutants K620I and R623/K624I, reduced binding of R619I and R626L, and little effect on binding of R623L. Sequence alignment of ICP0 homologues from several different alpha herpesviruses show almost complete conservation within RKCARKT (Fig 3C). In order to further delineate the importance of ^619^RK^620^ and ^623^RK^624^ within ICP0 residues 615–629, mutations were tested for interaction with Ubl123 using GST pull-down assays. All of the GST-ICP0 fusions containing a mutation in either of the K residues (K620A, K624A, R619A/K620A, R623A/K624A, K620A/K624A and RRKK) lacked detectable binding to Ubl123, confirming that loss of only one of the K residues is enough to disrupt binding (Fig 3D). The GST-ICP0 fusions containing a mutation in R residues retained binding to Ubl123 (Fig 3D) indicating that these R residues are not essential for the USP7-ICP0 peptide interaction in agreement with the structural analysis.

Fluorescence polarization was used to quantify the dissociation constant (K_D_) between USP7 and the ICP0 peptide. The concentration of the N-terminal FITC containing ICP0 peptide was kept constant at 40 nM and increasing amounts of protein were added (up to 200 μM). The dissociation constants were calculated to be 1.6 ± .2 μM for Ubl123, 0.4 ± 0.1 for USP7-CTD and 3.2 ± 0.6 μM for USP7-FL (Fig 3E). USP7-NTD and Ubl345 did not show any polarization indicating that they do not interact with the ICP0 peptide. The dissociation constants for the interaction between Ubl123 mutants (E759A, K762A, K764A and K762A/K764A) and ICP0 peptide were calculated to be above 100 μM indicating very weak binding (Fig 3E). The saturation curves are shown in S3 Fig.

Several key binding residues on Ubl123 were identified following the structural analysis of the Ubl123-ICP0 peptide complex including E759, D762 and D764. GST pull-down assays were used to assess the binding between ICP0 and Ubl123 or USP7-CTD mutants corresponding to these residues. Binding was abolished between ICP0 615–629 and Ubl123 or USP7-CTD mutants E759A, D762A, D764A and D762A/D764A indicating that each of these residues are important in complex formation between USP7 and the ICP0 peptide (Fig 3F and 3G).

To further assess the importance of the interaction between Ubl2 of USP7-CTD and ICP0, GST pull-down assays were performed with a longer ICP0 peptide corresponding to residues 594–775 (Fig 1A). As shown in Fig 3H, interaction was observed between GST-ICP0 594–775 and WT USP7-CTD but not D762R/D764R USP7-CTD. These binding assays indicate the importance of the Ubl2 aspartate residues in mediating interaction with ICP0 in the context of a longer fragment of ICP0.

### Using the USP7 binding motif of ICP0 to identify the mechanism of the USP7-GMPS interaction

**Fig 4:**
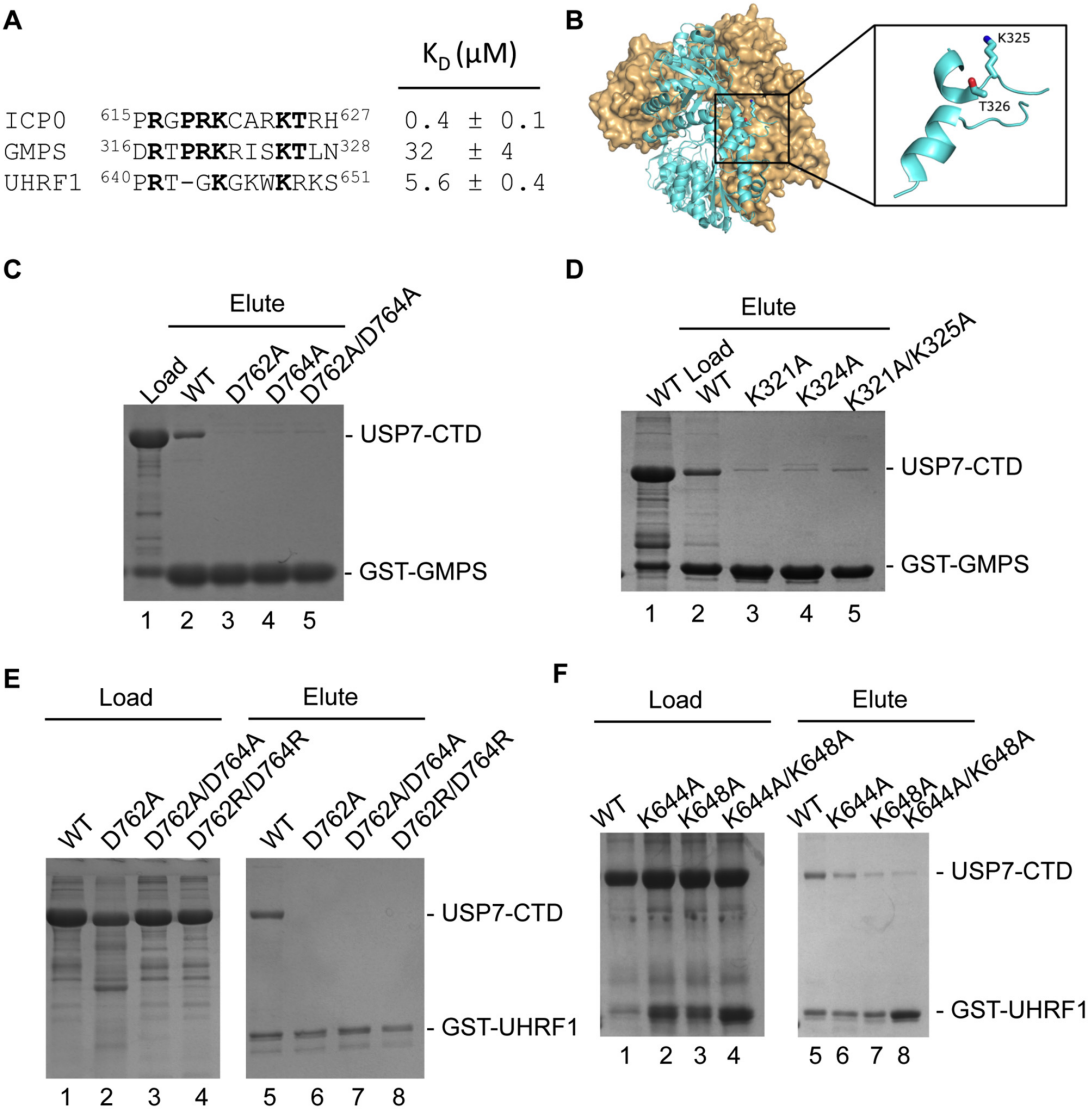
USP7-CTD interacts with GMPS and UHRF1 peptides. (A) Alignment and dissociation constants of ICP0, GMPS and UHRF1 peptides. (B) Location of the KxxxK motif (^316^DRTPRKRISKTLN^328^) within a disordered loop on the GMPS crystal structure (PDB ID 2VXO). (C) Coomassie stained gel of GST pull-down assays with WT or mutant USP7-CTD (D762A, D764A and D762A/D764A) and GST-GMPS peptide. Lane 1 USP7-CTD and GST-GMPS peptide load, lanes 2–5 are the eluted fractions with WT and mutant USP7-CTD. (D) Coomassie stained gel of GST pull-down assays with WT USP7-CTD and mutant GST-GMPS peptides. Lanes 1 (load) and 2 (elute) USP7-CTD and WT GST-GMPS, lanes 3–5 are the eluted fractions with GST-GMPS mutants (K321A, K325A and K321A/K325A). (E) Coomassie stained gel of GST pull-down assays with WT or mutant USP7-CTD (D762A, D762A/D764A and D762R/D764R) and GST-UHRF1 peptide. Lanes 1–4 are the loaded fractions with WT and mutant USP7-CTD. Lanes 5–8 are the eluted fractions with WT and mutant USP7-CTD. (F) Coomassie stained gel of GST pull-down assays with WT USP7-CTD and mutant GST-UHRF1 peptides. Lanes 1–4 are the loaded fractions with WT and mutant (K644A, K648A and K644A/K648A) GST-UHRF1. Lanes 5–8 are the eluted fractions with WT and mutant (K644A, K648A and K644A/K648A) GST-UHRF1. In all instances, approximately 1–2% of the input and 2.5% of the eluate is loaded on the gels.

GMPS interacts with USP7 within a region encompassing CAT-Ubl123 [15]. Once the crystal structure of the Ubl123-ICP0 peptide complex was determined and the importance of the K residues in ICP0 was established, we searched the GMPS sequence looking for a similar motif. A sequence that closely resembles the ICP0 KxxxK motif was located between residues ^316^DRTPRKRISKTLN^328^ (Fig 4A). The sequence is within a disordered loop on the surface of GMPS and so would be accessible for protein interactions (Fig 4B). GST pull-down assays were used to test whether this GMPS peptide interacted with USP7-CTD. As shown in Fig 4C, the GST-GMPS peptide interacted with USP7-CTD. These results confirm that GMPS residues ^316^DRTPRKRISKTLN^328^ bind USP7. The dissociation constant (K_D_) between USP7 and this GMPS peptide was also determined using fluorescence polarization. Similar to the ICP0 binding assay, the concentration of the N-terminal FITC containing GMPS peptide was kept constant at 40 nM and increasing amounts of USP7-CTD were added. The dissociation constant was calculated to be 32 ± 4 μM for USP7-CTD (Fig 4A). The saturation curve is shown in S3 Fig.

The importance of USP7 residues D762 and D764 for binding GMPS was also assessed using GST pull-down assays. D762A, D764A and D762A/D764A mutations of USP7-CTD disrupted binding to GST-GMPS, indicating that each of these residues are important in complex formation between USP7 and GMPS (Fig 4C). These results mimic the effects on ICP0 binding and confirm that the GMPS peptide binds USP7 through the Ubl2 binding pocket.

Finally, we examined the importance of the K residues in the USP7-binding peptide of GMPS for binding USP7-CTD. GST-GMPS peptides with either or both of the K residues mutated to A were assayed for binding USP7-CTD (Fig 4D). All of these mutations disrupted GMPS peptide binding to USP7, confirming the importance of the K residues in the GMPS-USP7 interaction and indicating that GMPS and ICP0 bind Ubl2 using a similar mechanism.

### UHRF1 also binds the Ubl2 binding pocket of USP7

A previous study indicated that, like ICP0 and GMPS, UHRF1 binds USP7 through its CTD (Ma et al, 2012). Given the importance of the KxxxK motif in ICP0 and GMPS for USP7-CTD binding, we examined the UHRF1 sequence for such a motif and identified ^642^TGKGKWKR^649^ (Fig 4A). Binding of the UHRF1 peptide to USP7-CTD was verified by fluorescence polarization assays, giving a dissociation constant (K_D_) of 5.6 ± 0.4 μM (Fig 4A). The saturation curve is shown in S3 Fig. A competitive binding assay was used to establish whether the UHRF1 and ICP0 peptides were competing for the same binding site on Ubl2. This assay was performed by measuring the fluorescence polarization with increasing amounts of unlabeled ICP0 while keeping both USP7-CTD and FITC-UHRF1 constant. The K_i_ was calculated to be 20.6 μM. When comparing this value to the dissociation constant obtained for interaction between USP7-CTD and UHRF1, this higher value suggests that the ICP0 peptide would be able to displace (compete with) bound UHRF1 peptide at the Ubl2 binding site. This competition was confirmed by the competition binding experiment shown in S3 Fig.

Similarly, an interaction between the UHRF1 peptide and USP7-CTD was detected in GST pull-down assays and this interaction was disrupted by D762A, D762A/D764A and D762R/D764R mutations in USP7 (Fig 4E), confirming binding to the Ubl2 binding pocket. In addition, mutagenesis of lysine residues K644 and K648 forming the KxxxK motif compromised binding to USP7-CTD (Fig 4F). The results confirm that UHRF1 can bind the same USP7 binding pocket as ICP0 and GMPS.

### Contributions of the Ubl2 binding pocket of USP7 for ICP0, GMPS and UHRF1 interactions in human cells

**Fig 5:**
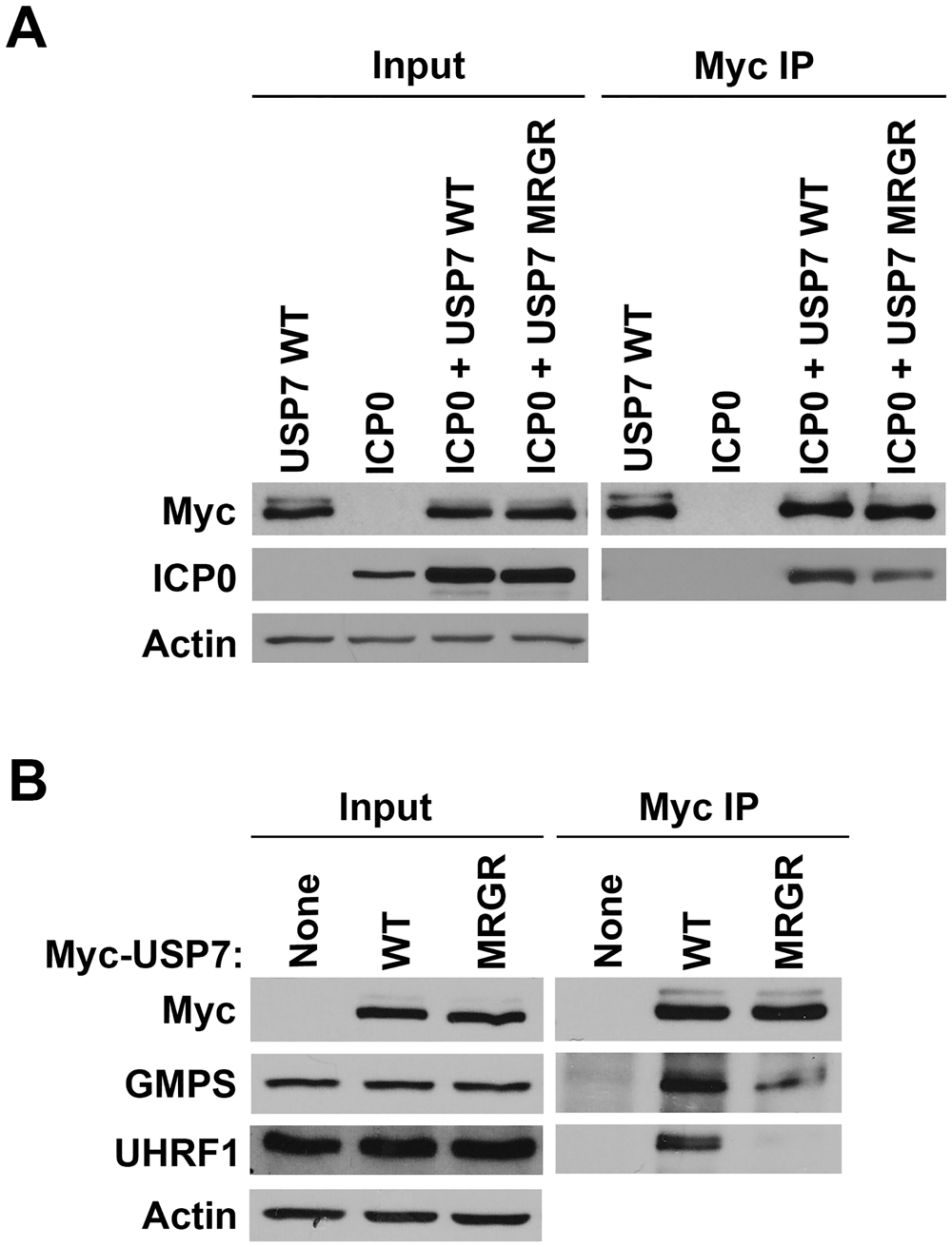
The D762R/D764R (MRGR) mutation disrupts ICP0, GMPS and UHRF1 binding to USP7 in human cells. (A) 293T cells were transfected with plasmid expressing myc-tagged WT or D762R/D764R (MRGR) USP7 and ICP0 or with WT USP7 or ICP0 expression plasmids alone. USP7 was precipitated with anti-myc antibody and recovered proteins were detected by Western blotting with antibodies against myc and ICP0. (B) 293T cells were transfected with plasmid expressing myc-tagged WT or D762R/D764R (MRGR) USP7 or with empty plasmid (None). USP7 was precipitated with anti-myc antibody and recovered proteins were detected by Western blotting with antibodies against myc, GMPS or UHRF1.

While our *in vitro* data indicate that ICP0, GMPS and UHRF1 can contact USP7 through the Ubl2 binding pocket, we wanted to determine the degree of importance of this interaction in human cells in the context of full length USP7. To this end we expressed myc-tagged USP7 with or without a D762R/D764R mutation in the Ubl2 binding pocket (referred to as MRGR in Fig 5) in 293T cells along with ICP0, immunoprecipitated USP7 by virtue of the myc tag and examined recovery of ICP0. As shown in Fig 5A, the recovery of ICP0 was decreased (2-fold in multiple experiments) but not abrogated by the D762R/D764R mutation, suggesting that, while these Ubl2 contacts contribute to USP7 binding, additional contacts of ICP0 with USP7 must also occur. Such additional contacts are also suggested by the fact that both WT USP7 and the D762R/D764R mutant had a stabilizing effect on ICP0 (compare input levels of ICP0 with and without USP7). We also expressed the myc-tagged WT and mutant USP7 proteins in the absence of ICP0, immunoprecipitated USP7 and examined recovery of endogenous GMPS and UHRF1 by Western blotting. As shown in Fig 5B, both GMPS and UHRF1 were efficiently recovered with WT USP7 but recovery was significantly decreased by the D762R/D764R mutation. Quantification from multiple experiments indicated that the D762R/D764R mutation reduced GMPS binding to 35% (± 16%) and UHRF1 binding to 21% (± 6%) relative to recovery with WT USP7. The results confirm the importance of the Ubl2 binding pocket for USP7 interaction with ICP0, GMPS and UHRF1.

### A flexible hinge between Ubl2 and Ubl3

**Fig 6:**
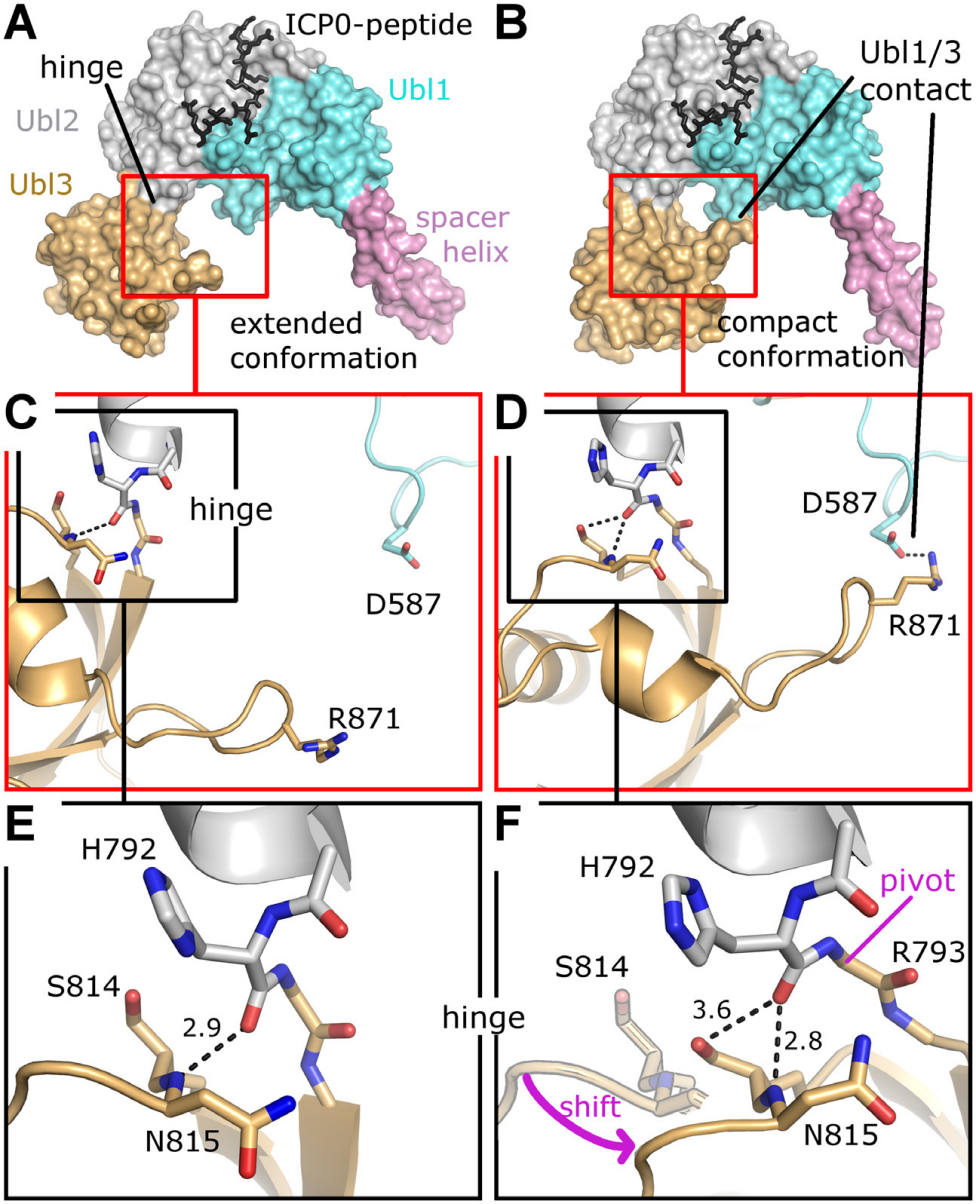
Two conformational states of Ubl123. (A) Extended conformation as observed in Se-Ubl123 (this work) and native USP7-CTD (PDB ID 2YLM). (B) Compact conformation observed for native Ubl123 (this work). (C) Details of contact formation between Ubl1 and Ubl3. (D) Details of contact formation between Ubl1 and Ubl3. (E) Details of the Ubl2-Ubl3 hinge. In F, the extended conformation is partially given for direct comparison; note that S814 is now occupying the former position of the backbone nitrogen atom of N815. A pink arrow indicates the shift from the extended to the compact conformation. The entire Ubl3 domain appears to rotate against Ubl12, the pivot point being between residue H792 and R793. Dashed lines indicate hydrogen bonds and salt-bridges, distances are given in Å. (F) Details of the Ubl2-Ubl3 hinge. In F, the extended conformation is partially given for direct comparison; note that S814 is now occupying the former position of the backbone nitrogen atom of N815. A pink arrow indicates the shift from the extended to the compact conformation. The entire Ubl3 domain appears to rotate against Ubl12, the pivot point being between residue H792 and R793. Dashed lines indicate hydrogen bonds and salt-bridges, distances are given in Å.

The crystal structure of all five C-terminal Ubl domains shows an extended conformation of Ubl123 [15], matching the conformation of our Se-Ubl123 structure (Fig 6A). Interestingly, native Ubl123 crystallized in a previously unknown compact conformation with a direct contact between Ubl1 and Ubl3 (Fig 6B). A superposition of all available USP7 fragments containing Ubl123 shows two distinct orientations of Ubl3 compared to Ubl12 with a 35 degree motion and a pivot at residue H792 (S4 Fig). Closer inspection of the residues within the hinge reveals that the change in orientation is originating in the connection between Ubl2 and Ubl3 (Fig 6C and 6D), involving a change in the hydrogen bond network of residues H792, S814 and N815 (Fig 6E and 6F). The only strong hydrogen bond between Ubl2 and Ubl3 is formed between the protein backbone of residues H792 and N814. In the compact conformation S814 has moved to the former position of N815, thereby tilting the entire Ubl3 domain against Ubl12. The compact conformation appears to be stabilized by a salt bridge between R871 of Ubl3 and D587 of Ubl1 (Fig 6D). The importance of a hinge between Ubl2 and Ubl3 becomes evident in a more complete USP7 model, whose generation and impact are discussed below.

### Allosteric USP7 regulation in the light of a nearly complete three-dimensional model

**Fig 7:**
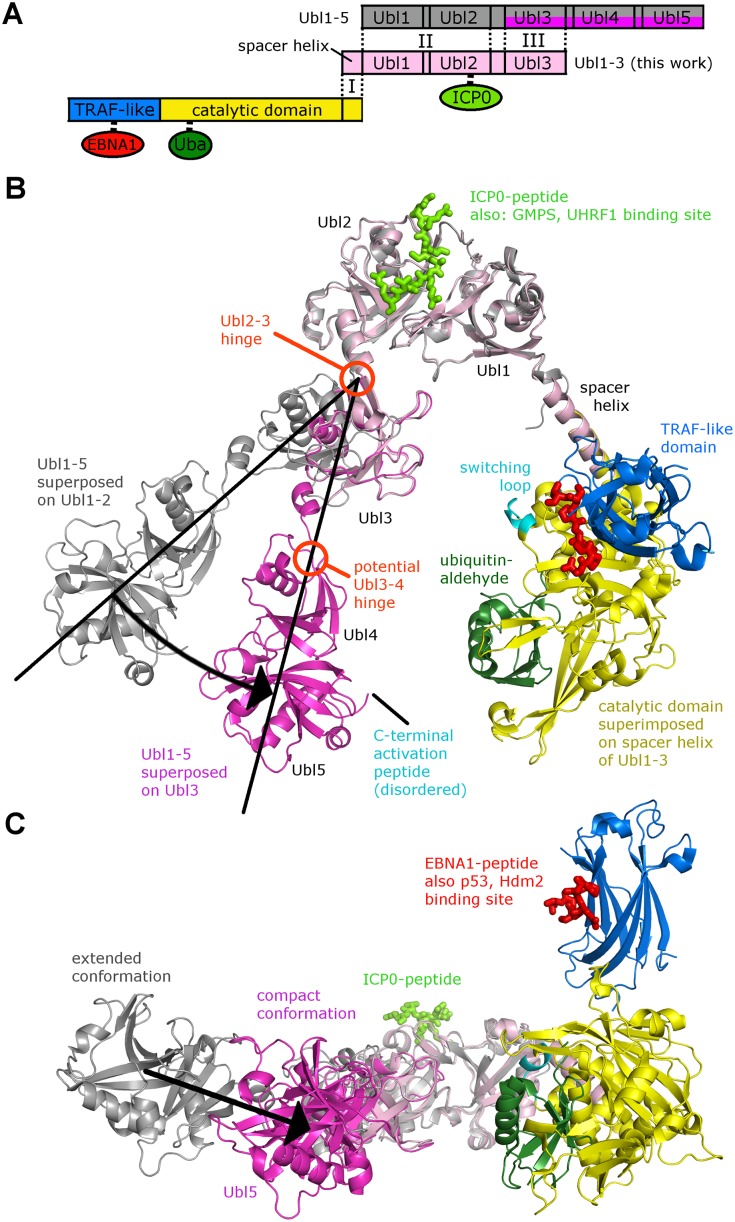
Three-dimensional model of full-length USP7 suggests back folding of Ubl45 onto the CAT domain. (A) Model generation: I. The crystal structure containing both NTD and CAT domain (blue/yellow, PDB ID 2F1Z) is superposed onto the spacer helix of Ubl123 (salmon, this work); II. The crystal structure of CTD (cyan, PDB ID 2YLM) is superposed onto Ubl1 and Ubl2 of Ubl123; III. The same crystal structure of CTD (magenta, omitting Ubl12 for clarity) is superposed onto Ubl3 of Ubl123 to generate an alternate compact conformation. Separate structures of the NTD containing EBNA1-peptide (red, PDB ID 1YY6) and the CAT domain containing ubiquitin-aldehyde (dark green, PDB ID 1NBF) are superposed onto the corresponding domains. (B) USP7 model in cartoon representation. Domains are shown in the same color-coding as in A. The Ubl2-Ubl3 hinge causes two conformations; a black arrow indicates the corresponding shift. (C) Side-view with Ubl5 and the catalytic domain in the foreground.

Crystal structures for various fragments of USP7 have been determined, including CAT, NTD, NTD-CAT and CTD [9, 15, 41, 42]. However, the spatial assembly of the entire 1102 amino-acid protein has remained elusive, obstructing insights into the regulation of the enzyme. The problem in building a full-length USP7 model from several domains is the lack of overlap between CAT and Ubl12. There are five rigid structural domains in USP7 (NTD, CAT, Ubl12, Ubl3, Ubl45, see Fig 7A), but the degree of flexibility between these five domains remained unclear. Our Ubl123 construct included the spacer helix (residues 535–560), which connects CAT with the Ubls. The spacer helix is partly present in several crystal structures of CAT, adopting a stable orientation (S4 Fig). Our crystallographic studies reveal a very similar orientation of the spacer helix in four crystallographically independent chains (S4 Fig), suggesting that this helix provides a preferred orientation between CAT and Ubl12, significantly minimizing the spectrum of possible full-length conformations of USP7. The overlapping spacer helix together with the CTD now allows the generation of a nearly complete three-dimensional model of USP7 (Fig 7B and S4 Fig), only missing some disordered N- and C-terminal residues. The resulting model of CAT-CTD is somewhat V-shaped, and intriguingly, the hinge between Ubl2 and Ubl3 (described above, Fig 6) affects the angle between the two arms and thereby the distance between Ubl45 and CAT (Fig 7B and 7C and S4 Fig). This suggests that the C-terminal Ubl domains fold back onto the CAT, which has also been suggested by a recent SAXS analysis of CAT-CTD [15]. The catalytic triad in USP7 is only properly aligned and functional in the ubiquitin-bound form [42], and ubiquitin-binding is promoted by Ubl45 [15], so the distance between these two sites is very likely a critical factor in deubiquitination activity of USP7. Catalytic activity and ubiquitin-binding is also related to the conformation of the so called “switching loop” located in the CAT domain (Fig 7B), and it has been proposed that the C-terminal activation peptide located just beyond Ubl45 has to come into contact with this loop for auto-activation [15]. In the compact conformation of Ubl123 (Fig 7B), the distance between the catalytic domain and Ubl45 is drastically reduced from 75 Å (extended conformation) to 50 Å. We assume that this distance is further minimized by a change in the orientation between Ubl3 and Ubl45 (see proposed Ubl3/4-hinge in Fig 7B), which would bring the activation peptide in contact with the switching loop.

## Discussion

USP7 plays a critical role in the regulation of many cellular processes, and viruses like HSV-1, EBV and KHSV hijack USP7 for their purposes. Despite the growing list of C-terminal domain interacting proteins, little is known about the molecular mechanisms of CTD interactions. Our study reveals a novel binding site in the C-terminal ubiquitin-like domains of USP7 that is distant to our previously characterized binding site for p53, HDM2, MCM-BP and EBNA1 at the N-terminal TRAF-like domain. Our structural analysis shows that an ICP0 peptide binds to a negatively charged region on Ubl2 via its ^618^PRKCARKT^625^ sequence thus revealing the USP7-CTD interaction mechanism. ICP0 residues K620 and K624 appear to be important for mediating the interaction as they form direct contacts with residues D762 and D764 in Ubl2 of USP7. Mutagenesis of the lysine residues in the ICP0 peptide resulted in decreased binding, revealing that these residues were essential for interaction with USP7. This allowed us to identify a KxxxK motif within a poly-basic region on the ICP0 peptide that interacts preferentially with the acidic patch on Ubl2 of USP7. In this respect, the positive charge of the poly-basic region might be necessary for a long range electrostatic interaction between the two proteins, whereas the KxxxK motif most likely allows specific binding to the buried aspartate residues of Ubl2. This novel USP7-CTD binding motif allowed us to identify similar motifs in GMPS (^319^PRKRISKT^326^) and UHRF1 (^642^TGKGKWKR^649^) and should be useful for the identification of Ubl2 binding sites in other USP7-CTD interacting proteins.

The identified GMPS and UHRF1 motifs, ^319^PRKRISKT^326^ and ^642^TGKGKWKR^649^, bind to the same region on Ubl2 of USP7 as the ICP0 peptide. Similar to ICP0, mutagenesis of either or both of the lysine residues in GMPS (K321 and K325) and UHRF1 (K644 and K648) decreased their interaction with USP7 *in vitro* indicating their mode of binding was mediated via similar contacts with Ubl2 of USP7. Our study also identified residues D762 and D764 on Ubl2 as making important contacts with USP7 binding partners that interact via the C-terminal ubiquitin-like domains. Mutagenesis of both acidic residues in USP7 resulted in decreased binding to ICP0, GMPS and UHRF1 *in vitro* as well as *in vivo*.

Intriguingly, the KCARK USP7 binding motif is present in many but not all ICP0 proteins. For example this sequence is absent in ICP0 homologues from equine and bovine alphaherpesviruses. While ICP0 proteins from bovine and equine viruses were found to have many properties in common with HSV-1 ICP0, unlike HSV-1 ICP0, neither equine nor bovine ICP0 relocalized USP7 [43, 44]. This in combination with our current results, indicates the importance of the KxxxK binding motif for USP7 binding.

Viral proteins often make use of existing cellular mechanisms and it appears that ICP0 has evolved to target USP7 by using the GMPS/UHRF1 binding site on Ubl2. Moreover viral proteins typically bind cellular proteins with higher affinity than the endogenous binding partners, thereby blocking the endogenous interaction. This is exemplified with the N-terminal TRAF domain of USP7, as viral proteins EBNA1 and vIRF4 bind with at least a 10-fold higher affinity to the TRAF binding pocket than cellular substrate proteins [9, 25]. The K_D_s determined for ICP0, GMPS and UHRF1 binding to USP7-CTD suggest a similar scenario for the CTD binding pocket; namely that ICP0 might effectively compete for the CTD binding site, limiting endogenous interactions that occur through this site. In fact, our peptide binding assays demonstrate that the ICP0 peptide readily competes with the UHRF1 peptide for binding to Ubl2.

While ICP0, GMPS and UHRF1 are unrelated proteins, all three use the same Ubl2 binding site to interact with USP7. However, the fact that these interactions were decreased but not abrogated by the Ubl2 D762R/D764R mutation suggest that additional contacts with USP7 also occur. Whether or not the additional contacts of ICP0, GMPS and UHRF1 are the same or different remains to be determined. These three proteins also appear to interact with USP7 for different purposes. ICP0 uses USP7 for its own stabilization [35] and also dampens immune responses by translocating USP7 to the cytosol where it deubiquitinates NF-kB related signaling proteins [40]. The USP7-GMPS deubiquitinating complex regulates p53 levels [20], as well as chromatin structure via H2B deubiquitination [17–19]. In a ternary complex with UHRF1 and DNMT1, USP7 affects DNA methylation [1]. Intriguingly, the identified USP7 binding region in UHRF1 has been recently described as an internal binding peptide in UHRF1 affecting the mode of histone H3 binding [45]. In free UHRF1, the so-called polybasic region (PBR: ^641^RTGKGKWKRKSAGGGPS^657^) binds to a grove in the tandem tudor domain blocking histone H3 trimethylated-K9 (H3K9me3) binding, whereas in complex with phosphatidylinositol 5-phosphate (PI5P) the PBR is employed in the UHRF1-PI5P contact formation, allowing H3K9me3 binding of UHRF1. Thus, in addition to internal binding to the tandem tudor domain, the PBR region in UHRF1 has at least two interacting partners, USP7 and PI5P, suggesting an intricate regulatory mechanism within variable multi-protein complexes.

Our crystal structures reveal an alternative compact conformation of Ubl123, indicating a flexible hinge between Ubl2 and Ubl3. It has already been proposed that the C-terminal Ubl45 domain, together with the “activation peptide” are required to activate the catalytic triad and stabilize ubiquitin binding to the catalytic domain [15], which is allosterically regulated by GMPS [15]. Our multi-domain model of USP7 together with the identified hinge between Ubl2 and Ubl3 suggest that Ubl45 can indeed come into contact with the catalytic domain. The binding site at Ubl2 offers GMPS a formidable position to control the conformation of the C-terminus relative to the catalytic domain. Our full-length model also suggests proximity between proteins bound to the TRAF-like domain and the C-terminal Ubl domains. Both p53 and its negative regulator Hdm2 contain a second binding site within USP7 that has been mapped to residues 801–1050 corresponding to Ubl45 [24, 46], suggesting both p53 and Hdm2 have multi-domain interaction with USP7. It is possible that a similar scenario exists for the ICP0-USP7 interaction since the D762R/D764R USP7 mutation decreased but did not abrogate USP7 binding in human cells. The parallels between GMPS and ICP0 binding to USP7 raise the possibilities that ICP0 might mimic GMPS in stimulating USP7 activity or alternatively that it might interfere with GMPS stimulation of USP7 activity by competing with GMPS for USP7. These will provide important topics for future study. To date we have tested whether the Ubl2-binding peptide of ICP0 affected ubiquitin cleavage by USP7 but have not detected an effect (data not shown).

Our structural analysis revealed a novel binding site at the C-terminal ubiquitin-like domains used by both viral and cellular USP7 binding proteins and sheds light on the mechanistic principles of USP7 regulation, which affect apoptosis, cell cycle arrest, chromatin remodeling and immune responses. A precise understanding of USP7 regulation is necessary to target USP7 for the development of cancer therapeutics.

## Materials and Methods

### Expression plasmids and mutants

Fragments representing USP7-CTD Ubl domains were prepared using ligation independent cloning with In-Fusion (Clontech) in p15TV-L. The boundaries of the Ubl domain constructs are Ubl12 (535–776), Ubl123 (535–889), Ubl345 (776–1102), Ubl45 (886–1102) and USP7-CTD (535–1102). Plasmids expressing USP7-NTD, USP7, and myc-USP7 WT (pCANmycUSP7) were described previously [9, 47]. USP7 mutants E759A, D762A, D764A and D762A/D764A in Ubl123 were synthesized (Genscript) and inserted into the pET15b plasmid. USP7 mutants E759A, D762A, D764A, D762A/D764A and D762R/D764R in USP7-CTD were prepared by ACGT Corporation. The D762R/D764R mutant was then sub-cloned into wild-type pCANmycUSP7 for co-IP assays. The mammalian ICP0 expression construct pCI-110 ICP0 was obtained from Roger Everett [38].

### Protein purification

The USP7 domain constructs were expressed in *E*. *coli* BL21(DE3)mgk and full length USP7 was expressed in *Sf9* cells. Selenomethionine enriched protein was prepared using the methionine inhibition pathway in *E*. *coli* BL21(DE3)mgk. Cell pellets from various amounts of LB broth were resuspended with binding buffer (20 mM Tris pH 7.5, 500 mM NaCl, 5 mM imidazole containing phenylmethylsulfonyl fluoride and benzamidine) and lysed using sonication. The lysate was cleared via centrifugation and applied to nickel-NTA agarose beads. After extensive washing, the bound proteins were eluted in 20 mM Tris pH 7.5, 500 mM NaCl containing 500 mM imidazole. Size exclusion chromatography using Hi-Load Superdex 200 pg (16/60) on an AKTA Purifier (GE Healthcare) in 20 mM Tris pH 7.5 and 167 mM NaCl at 4°C was performed on all USP7 proteins.

### GST pull-down assays

The ICP0, GMPS and UHRF1 WT and mutant peptides were cloned into pGEX-2TK and expressed in *E*. *coli* BL21(DE3). GST-ICP0 594–775 was obtained from Roger Everett [48]. The GST-ICP0 or GST-GMPS or GST-UHRF1 or GST-ICP0 594–775 cell pellets from 500 ml of LB broth were re-suspended with PBS and lysed using sonication. The cleared lysates were applied to 500 μl of equilibrated glutathione-sepharose beads and incubated with rocking for 60 min at 4°C. Unbound proteins were removed by washing with PBS. Bound proteins were eluted with 50 mM Tris pH 8.0 and 100 mM NaCL containing 30 mM reduced glutathione. Equal amounts (250 μg) of the various USP7 proteins were incubated with 250 μg of the GST-ICP0 or GST-GMPS or GST-UHRF1 or GST-ICP0 594–775 peptides in 50 mM Tris pH 8.0, 100 mM NaCl, 5 mM DTT, 5% glycerol, 0.1 mM Benzamidine and 0.05 mM PMSF, applied to equilibrated glutathione-sepharose beads and incubated overnight with rocking at 4°C. After extensive washing, bound proteins were eluted with 50 mM Tris pH 8.0 and 100 mM NaCl containing 30 mM reduced glutathione.

### Fluorescence polarization assays

USP7 proteins were titrated (0 to 200 μM) with N-terminally labeled FITC-ICP0, FITC-GMPS or FITC-UHRF1 peptides (synthesized by CanPeptide, 40 nM) in 0.01% Triton X-100, 150 mM NaCl, 20 mM Tris pH 7.5. Polarized emission of FITC-ICP0 or FITC-GMPS or FITC-UHRF1 was measured with a Synergy H4 Hybrid Multi-Mode Microplate Reader (BioTek) with the excitation wavelength set at 485 nm and the emission wavelength set at 528 nm. The competition binding assay was performed with a fixed USP7-CTD concentration (8 μM), 40nM FITC-UHRF1 and increasing concentrations of unlabeled ICP0 (0 to 2500 μM). The polarization data were plotted and analyzed using GraphPad Prism version 5.0 to calculate dissociation (K_D_) and inhibition (K_i_) constants. These assays were repeated at least 3 times and used to calculate the average and standard deviation.

### Crystallization

Ubl123 (30 mg/ml) and ICP0 peptide (synthesized by Canpeptide Inc with N-terminal acetylation and C-terminal amidation) were mixed in a 1:2 ratio and allowed to interact for a few minutes before plating. Initial protein crystallization trials were set-up in 96-well plates (Douglas Instruments) with sparse matrix crystallization suites JCSGI-IV, JCSG+, PACT, PEGsII and ClassicsII (QIAGEN); positive hits were further optimized with an additive screen (Hampton Research). Native Ubl123-ICP0 peptide crystals were grown in hanging drops and equilibrated against 500 μl reservoir solution containing 16% (w/v) PEG 3000 and 100 mM sodium citrate at pH 6. The drops contained 2 μl protein solution, 2 μl reservoir solution and 0.4 μl 100 mM L-Proline as additive. Selenomethionine enriched Ubl123-ICP0 crystals were grown directly in 167 mM sodium chloride, 20 mM Tris pH 7.5, 5 mM β-mercaptoethanol and 10 mM betaine hydrochloride.

### Structure determination and refinement

Se-Ubl123 with ICP0 peptide: The native Ubl123 construct co-crystallized with ICP0 peptide in space group P3_2_21 and the crystals diffracted to 2.9 Å resolution, but molecular replacement with the corresponding domains in the C-terminal USP7 structure (PDB ID 2YLM) was unsuccessful. Therefore, a Se-MAD (multiwavelength anomalous dispersion with selenium) dataset was collected from SeUbl123-ICP0 at the CMCF beamline (08ID-1) at the Canadian Light Source [49] (Spacegroup P4_2_2_1_2, 3.4 Å resolution), integrated using XDS [50], and solved by SHELXD/SHELXE [51]. A long α-helix in the electron density map could be identified as the spacer helix, which was placed manually using a crystal structure of the catalytic domain which contained most of the spacer helix (PDB ID 1NB8). Ubl12 and Ubl3 (from PDB ID 2YLM) were separately placed in the electron density. The resulting model was refined in Refmac5 [52] (Ramachandran statistics: 2 outliers, 60 residues in allowed regions, 629 residues in preferred regions). Despite the lower resolution we included this structure because it showed Ubl123 in an extended conformation.

Native Ubl123 with ICP0 peptide: X-ray data was collected at 100K on a Rigaku MicroMax007 rotating anode diffractometer with Saturn 944+ CCD detector. The data were integrated using HKL2000 [53]. We used the model obtained by MAD phasing from Se-Ubl123 for molecular replacement with PHASER [54]. A search for 2 molecules in the asymmetric unit using Ubl12 including the spacer helix was successful. Ubl3 could be identified in the resulting electron density map for both chains, and was placed manually. The resulting model was then refined in Refmac5 [51] against 2.9 Å native data. It was found that the orientation of Ubl3 had changed, but that the orientation of the spacer helix remained constant, which might explain why the initial molecular replacement trial (using Ubl123 from PDB ID 2YLM without the spacer helix) had failed. Residual density indicated bound peptide adjacent to Ubl2 (S1 Fig). The peptide was modeled manually in Coot [55], and the electron density could be assigned unambiguously to the sequence of the ICP0 peptide. The final refinement statistics are listed in Table 1 (Ramachandran statistics: no outliers, 15 residues in allowed regions, 651 residues in preferred regions). Coordinates and structure factors have been deposited in the Protein Data Bank under accession codes 4WPH and 4WPI. Representations of proteins were generated with PYMOL [56].

### Cell lines and transfections

293T cells were grown in Dulbecco’s modified Eagle’s medium (DMEM) (Gibco) with 10% fetal calf serum. 293T cells at 70% confluence in a 10-cm-diameter dish were transfected with 10 μg pCANmycUSP7 expressing WT USP7 or D762R/D764R (MRGR) USP7 or with pcDNA3.1 (negative control), in each case using 20 μl PolyJet (SignaGen Laboratories) transfection reagent. Cells were transferred to a 15-cm-diameter dish 24 h post-transfection and harvested 48 h post-transfection. For experiments on USP7-ICP0 interactions, 293T cells were co-transfected with 1 μg pCANmycUSP7 expressing WT or D762R/D764R (MRGR) USP7 and either 1 μg pCI-110 ICP0 or 1 μg pcDNA3.1. For controls lacking USP7, 1 μg pCI-110 ICP0 was co-transfected with 1 μg pcDNA3.1. In each case 4 μl PolyJet (SignaGen Laboratories) transfection reagent was used.

### Western blotting and antibodies

Protein samples were subjected to SDS-PAGE and transferred to nitrocellulose. Membranes were blocked in 5% skim milk in Tris-buffered saline with 1% Tween (TBS-T) and then incubated with antibodies against ICP0 (H1A027 from Virusys Corporation; 1:5000 dilution), myc (sc-40 from Santa Cruz Biotechnology; 1:5000 dilution), NP95/UHRF1 (A301-470A from Bethyl Laboratories; 1:1000 dilution), GMPS (rabbit serum raised against full-length GMPS [17]; 1:5000 dilution) and actin (MA5-15739 from Pierce; 1:20,000 dilution). After washing, blots were probed with goat anti-mouse or goat anti-rabbit peroxidase (Santa Cruz Biotechnology; 1:5000 dilution) and developed using chemiluminescence reagents (Santa Cruz biotechnology).

### Immunoprecipitation of Myc-USP7

293T cells transfected as described above were washed in phosphate-buffered saline (PBS) and lysed on ice for 45 min in a 4x volume of RIPA buffer (50 mM Tris-HCl [pH 8], 150 mM NaCl, 0.1% sodium deoxycholate, 0.5% NP-40) with complete protease inhibitors (Sigma P8340), followed by centrifugation. 1 mg of each clarified lysate was incubated with 1 μg of anti-myc antibody for 1h, followed by incubation with 30 μl of A/G PLUS agarose resin (Santa Cruz Biotechnology) for 2 h at 4°C with end-over-end rotation. The resin was harvested by centrifugation, washed in RIPA buffer, and then boiled in SDS loading buffer. Immunoprecipitated proteins were separated by SDS-PAGE and analyzed by Western blotting, as described above.

## Supporting Information

S1 Fig Electron density maps of the ICP0 peptide bound to Ubl123. (A/C) Residual electron density calculated without the ICP0 peptide. (B/D) Regular electron density obtained after refinement of the final model containing both USP7 and ICP0 peptide. Both peptide-binding sites at chain A (pink) and chain B (light orange) are shown. For all cases native Ubl123 data was used (for statistics see Table 1).
(TIF)

S2 Fig Comparison between peptide-bound and apo Ubl123. (A) The peptide bound Ubl12 (this work) is compared to the apo-Ubl12 (PDB ID 2YLM). (B) Ubl12 with bound peptide. (C) Side-chains that have different orientations in the peptide bound and apo-form are labeled in red; both side-chain conformations are shown. (D) The involvement in peptide binding of side-chains undergoing a shift is shown.
(TIF)

S3 Fig Fluorescence polarization saturation curves. (A) FL-USP7 with ICP0 peptide. (B) WT and mutant Ubl123 with ICP0 peptide. (C) USP7-CTD with ICP0 peptide. (D) USP7-CTD with GMPS peptide. (E) USP7-CTD with UHRF1 peptide. (F) Competition between UHRF1 and ICP0 peptides with USP7-CTD.
(TIF)

S4 Fig Superposition of USP7 domains. (A) Superposition of C-terminal domains: five chains from three different crystal structures are superimposed onto Ubl12. A compact conformation (blue) is observed in the crystal structure of native Ubl123 in complex with ICP0 peptide (both in chains A and B). An extended conformation (green) is observed in the crystal structure of apo-USP7-CTD (PDB ID 2YLM) and Se-Ubl123 in complex with ICP0-peptide (both in Chains A and B). In these five chains the spacer helix has a similar orientation towards Ubl12. (B) Superposition of N-terminal domains: seven chains from three different crystal structures containing the catalytic domain with part of the spacer helix are superposed. Two chains also include the N-terminal TRAF-like domain. In all seven cases the spacer helix obtains a very similar orientation towards the catalytic domain. The overall conformation of the catalytic domain slightly changes when ubiquitin-aldehyde (shown in magenta/salmon) is bound, which is assumed to be part of the catalytic mechanism.
(TIF)
