# Supplementary figures and images for: Crystal Structure of USP7 Ubiquitin-like Domains with an ICP0 Peptide Reveals a Novel Mechanism Used by Viral and Cellular Proteins to Target USP7
Source: PLoS Pathog. 2015 Jun 5;11(6):e1004950. doi: 10.1371/journal.ppat.1004950 (PMC4457826; doi:10.1371/journal.ppat.1004950)

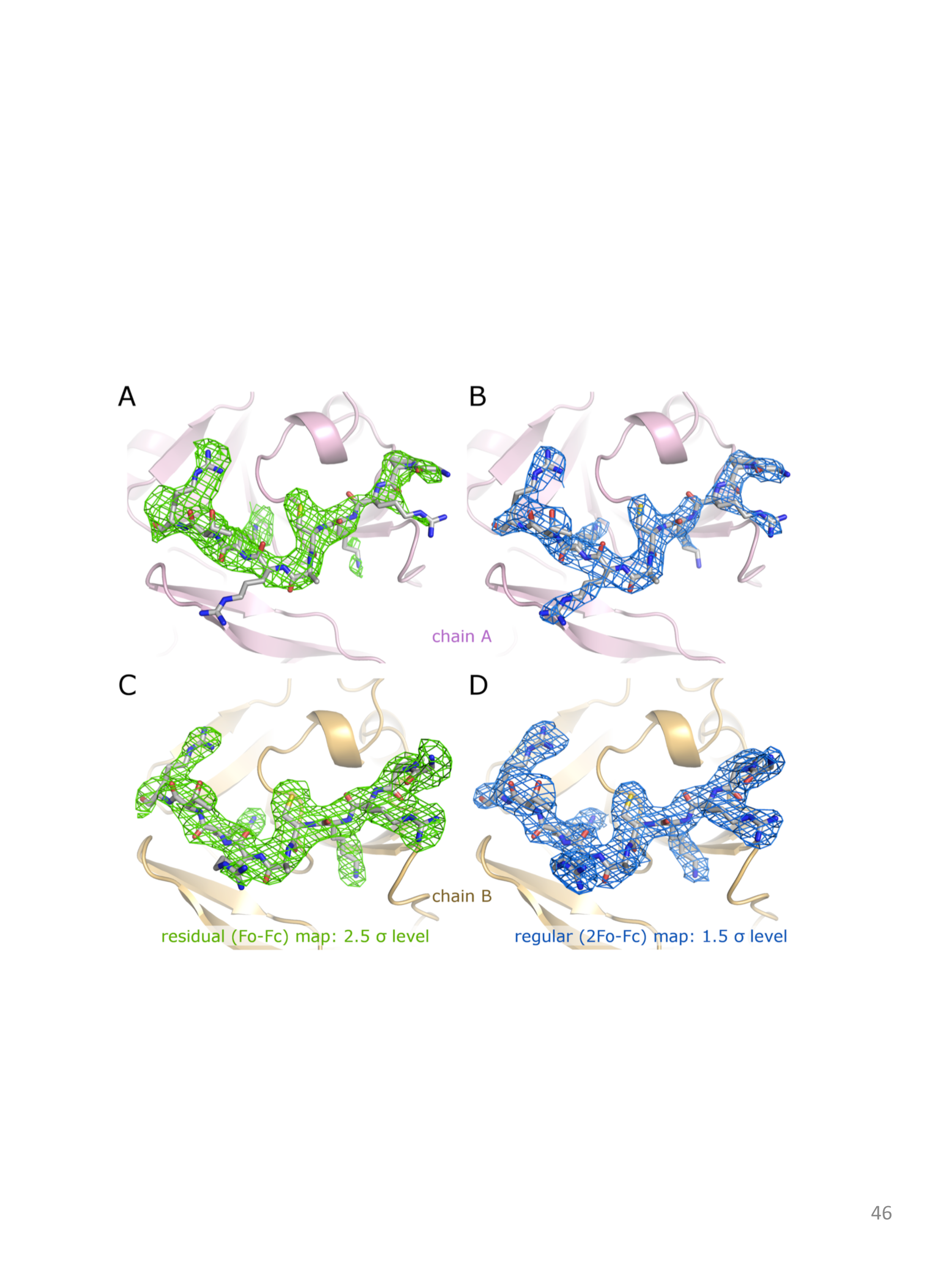

Supplement: S1 Fig — (A/C) Residual electron density calculated without the ICP0 peptide. (B/D) Regular electron density obtained after refinement of the final model containing both USP7 and ICP0 peptide. Both peptide-binding sites at chain A (pink) and chain B (light orange) are shown. For all cases native Ubl123 data was used (for statistics see Table 1). (TIF) [file ppat.1004950.s001.tif]

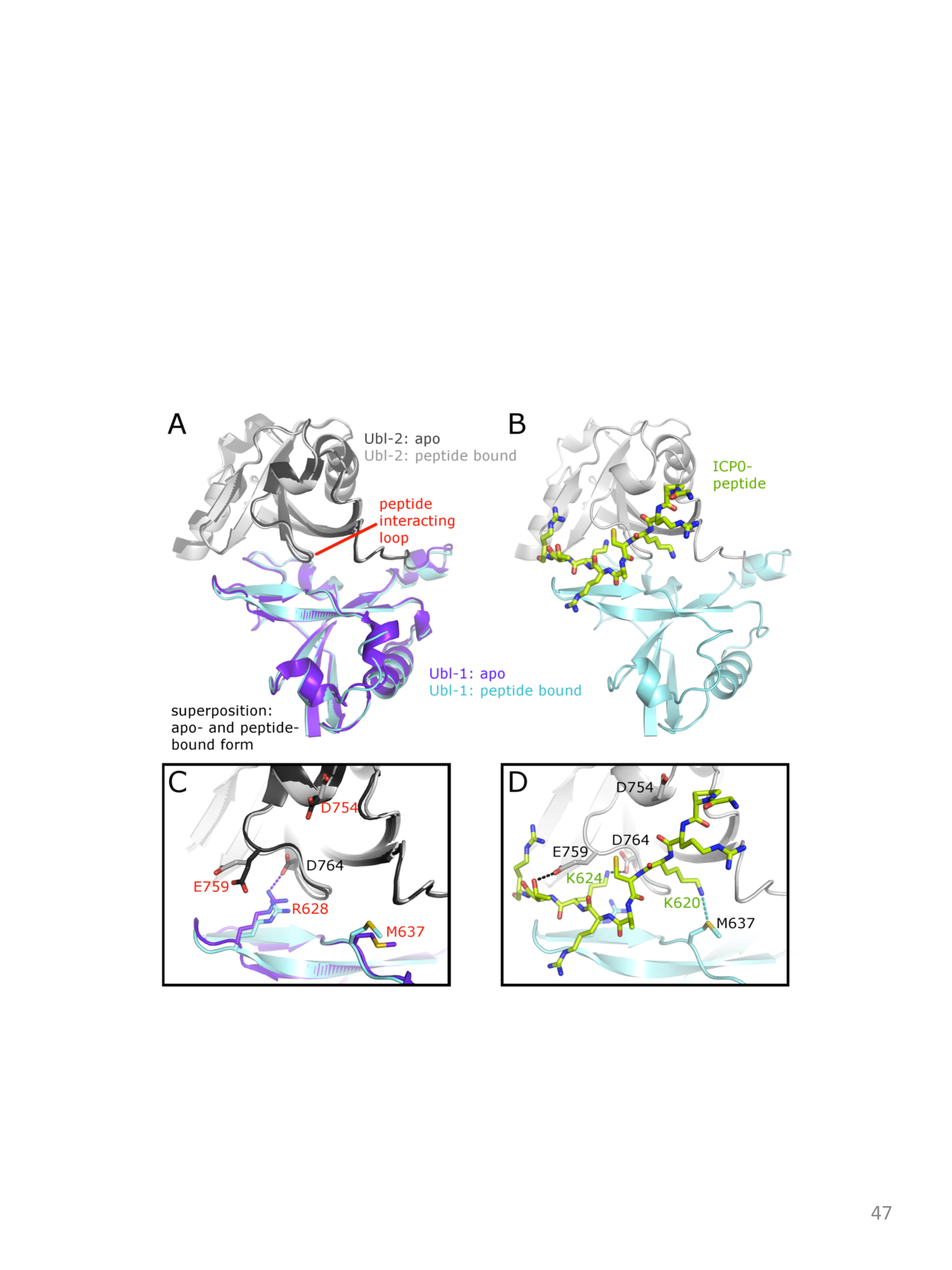

Supplement: S2 Fig — (A) The peptide bound Ubl12 (this work) is compared to the apo-Ubl12 (PDB ID 2YLM). (B) Ubl12 with bound peptide. (C) Side-chains that have different orientations in the peptide bound and apo-form are labeled in red; both side-chain conformations are shown. (D) The involvement in peptide binding of side-chains undergoing a shift is shown. (TIF) [file ppat.1004950.s002.tif]

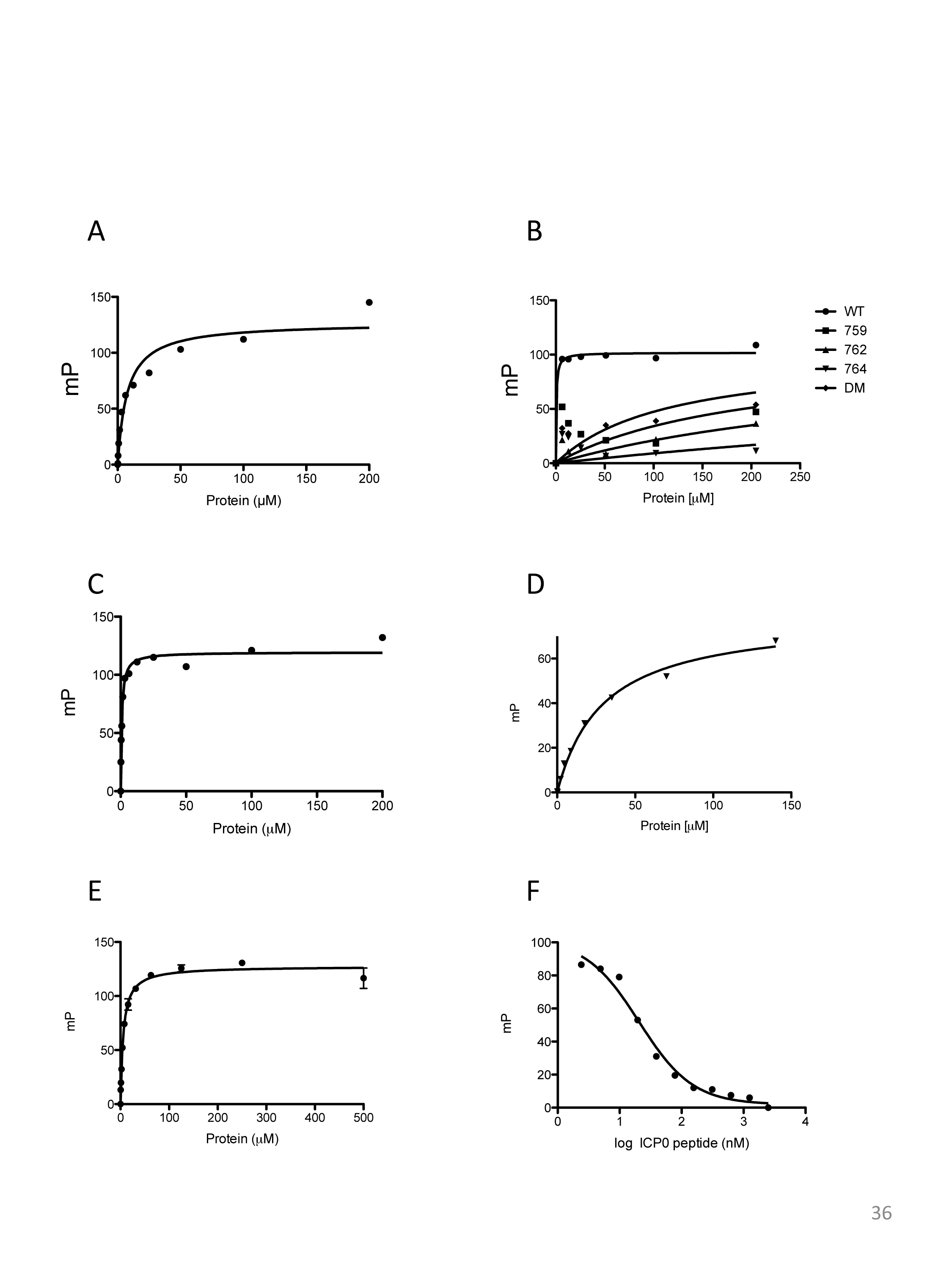

Supplement: S3 Fig — (A) FL-USP7 with ICP0 peptide. (B) WT and mutant Ubl123 with ICP0 peptide. (C) USP7-CTD with ICP0 peptide. (D) USP7-CTD with GMPS peptide. (E) USP7-CTD with UHRF1 peptide. (F) Competition between UHRF1 and ICP0 peptides with USP7-CTD. (TIF) [file ppat.1004950.s003.tif]

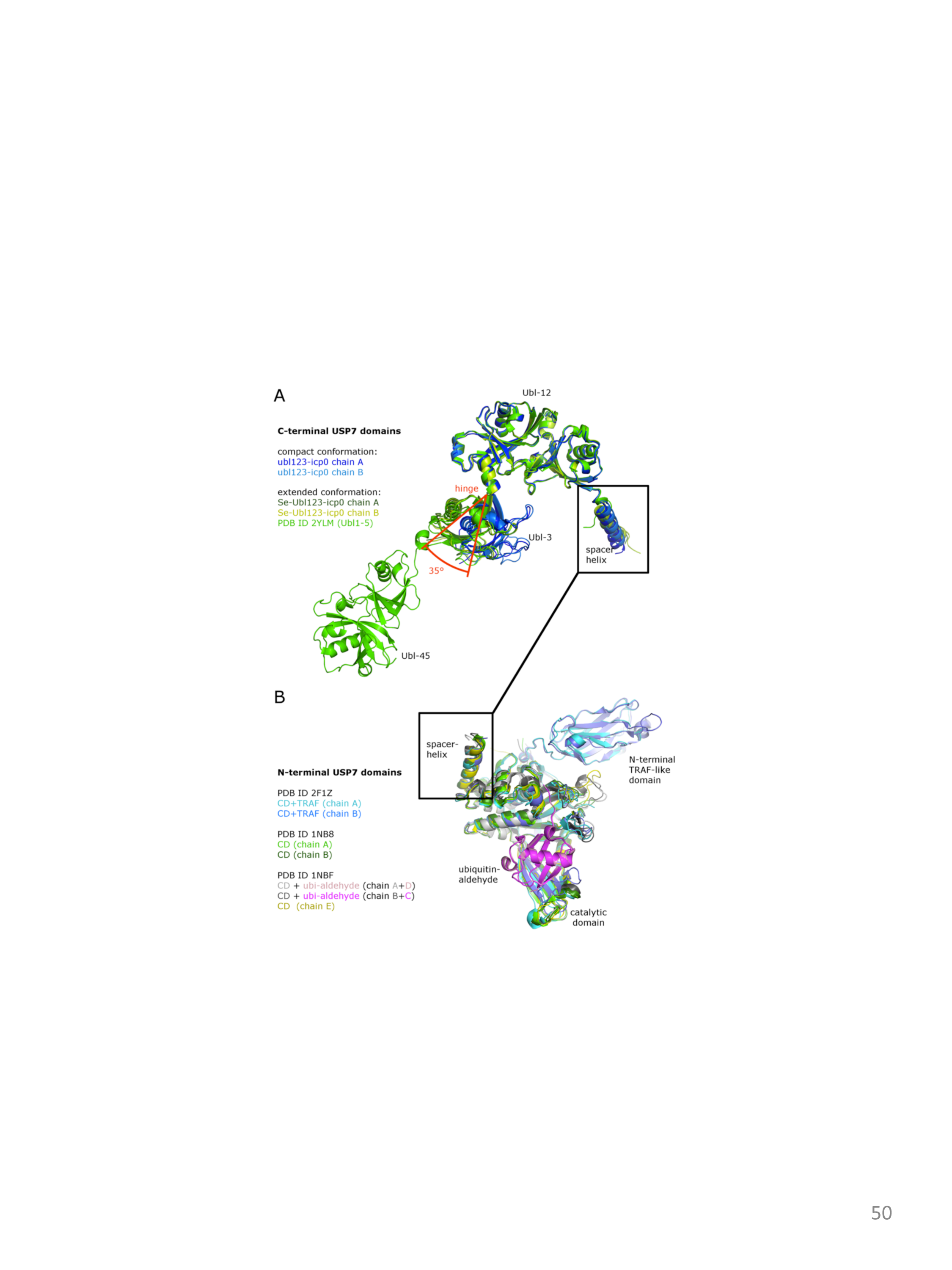

Supplement: S4 Fig — (A) Superposition of C-terminal domains: five chains from three different crystal structures are superimposed onto Ubl12. A compact conformation (blue) is observed in the crystal structure of native Ubl123 in complex with ICP0 peptide (both in chains A and B). An extended conformation (green) is observed in the crystal structure of apo-USP7-CTD (PDB ID 2YLM) and Se-Ubl123 in complex with ICP0-peptide (both in Chains A and B). In these five chains the spacer helix has a similar orientation towards Ubl12. (B) Superposition of N-terminal domains: seven chains from three different crystal structures containing the catalytic domain with part of the spacer helix are superposed. Two chains also include the N-terminal TRAF-like domain. In all seven cases the spacer helix obtains a very similar orientation towards the catalytic domain. The overall conformation of the catalytic domain slightly changes when ubiquitin-aldehyde (shown in magenta/salmon) is bound, which is assumed to be part of the catalytic mechanism. (TIF) [file ppat.1004950.s004.tif]
